# A pan-cancer analysis reveals role of clusterin (*CLU*) in carcinogenesis and prognosis of human tumors

**DOI:** 10.3389/fgene.2022.1056184

**Published:** 2023-01-04

**Authors:** Yizhe Fu, Qiao Du, Tiehan Cui, Yuying Lu, Guangliang Niu

**Affiliations:** ^1^ Department of Oral and Maxillofacial Surgery, the First Affiliated Hospital of Nanchang University, Nanchang, China; ^2^ Department of Stomatology, Beijing Integrated Traditional Chinese and Western Medicine Hospital, Beijing, China

**Keywords:** clusterin, tongue squamous cell carcinoma, pan-cancer analysis, prognosis, immune checkpoints

## Abstract

Clusterin (*CLU*) is a chaperone-like protein that has been demonstrated to have a direct relationship with cancer occurrence, progression, or metastasis. Clusterin was downregulated in tumor tissues using three datasets of tongue squamous carcinoma from the Gene Expression Omnibus. We further retrieved datasets from The Cancer Genome Atlas and Gene Expression Omnibus to thoroughly investigate the carcinogenic consequences of Clusterin. Our findings revealed that decreased Clusterin expression in malignancies was associated with a worse overall survival prognosis in individuals with multiple tumors; Clusterin gene deep deletions were found in almost all malignancies and were connected to most cancer patient’s prognosis, Clusterin DNA methylation level was dependent on tumor type, Clusterin expression was also linked to the invasion of cancer-associated CD8+ T-cells and fibroblasts in numerous cancer forms. Moreover, pathway enrichment analysis revealed that Clusterin primarily regulates biological processes such as cholesterol metabolism, phospholipid binding, and protein-lipid complex formation. Overall, our pan-cancer research suggests that Clusterin expression levels are linked to tumor carcinogenesis and prognosis, which contributes to understanding the probable mechanism of Clusterin in tumorigenesis as well as its clinical prognostic significance.

## 1 Introduction

Clusterin (*CLU*), also called apolipoprotein J, is a sulphated chaperone glycoprotein that plays a role in cell adhesion, membrane transport recycling, immune response, cell survival, and apoptosis (Trougakos and Gonos, 2002; Pucci and Bettuzzi, 2009; Yu and Tan, 2012). *CLU* is associated with lipid metabolism and Alzheimer’s disease (Yu and Tan, 2012). Several transcript variants of *CLU* resulting from alternative splicing have been documented, with the transcript encoding nuclear secretion and isoform being the most common. The disulfide-linked heterodimer 80 kDa (s*CLU*) functions in protein homeostasis with anti-apoptotic effects (Rohne et al., 2016). In contrast, the shortened nuclear version of *CLU* (n*CLU*) lacking exon two and ER signal peptide sequencing is pro-apoptotic (Bettuzzi and Rizzi, 2009; Prochnow et al., 2013). Pro-apoptotic *CLU* represses Bcl-xL by binding to Bax (Kim et al., 2012; Mustafi et al., 2017). Recently, s*CLU* overexpression increased autophagy *via* the AMPK/Akt/mTOR signaling pathway in oral squamous carcinoma cells, resulting in cell survival and protection against apoptosis (Naik et al., 2020). A comprehensive analysis of head and neck squamous cell carcinoma (HNSC) revealed that *CLU* targets miRNA-21 with proto-oncogenic properties. miRNA-21 overexpression leads to *CLU* downregulation, which sequentially stimulates tumor cell growth (Mydlarz et al., 2014).

We identified 238 upregulated, and 178 downregulated genes in squamous carcinoma (SCC) samples using three GEO datasets compared with normal tissues. Previous transcriptomic investigations suggested that 18 genes were downregulated in oral squamous cell carcinoma (OSCC) (Ye et al., 2008). We obtained 18 downregulated genes intersection and the down-regulated genes from the analysis of three GEO datasets and seven consistently downregulated genes, including *CLU* (*p <* 0.01). We then focused on *CLU* for further analysis.

s*CLU* is downregulated in oral cancer cell lines and tissues and exhibits tumor-suppressor-like actions (Kadam et al., 2021). Moreover, a series of studies revealed that *CLU* plays a crucial role in the lung (Panico et al., 2009), prostate (Rizzi and Bettuzzi, 2009), breast (Redondo et al., 2009), ovarian (Hoter and Naim, 2019), and colon cancers (Mazzarelli et al., 2009).

With the development of second-generation sequencing, bioinformatics tools for analyzing high-throughput expression data from cancer patients has become a prominent approach to cancer research. Multi-chip combined differential gene analysis, Gene Ontology (GO) and Kyoto Encyclopedia of Genes and Genomes (KEGG) enrichment analysis, and Cox regression analysis are all prevalent bioinformatics study approaches nowadays. In recent years, feature selection methods such as CHNMF and SDSPCA have also been proposed (Feng et al., 2019; Yu et al., 2021).

However, no pan-cancer study has evaluated the link between *CLU* expression and tumorigenesis/clinical prognosis. This study investigated the human *CLU* carcinogenic role in human cancers. Utilizing the TCGA project and GEO, pan-cancer *CLU* analyses were conducted, such as the characterization of the expression profile, prognostic value, DNA methylation, and key *CLU* cellular activities in various tumor types. *CLU* genetic modification status, prognostic value, and its association with immunological infiltration have also been studied in numerous cancer types. Our findings contribute to a better understanding of *CLU*’s role of *CLU* in the occurrence and prognosis of various malignant tumors.

## 2 Materials and methods

### 2.1 Data sets analysis and clinical sample characteristics from GEO database

Gene expression profiles for datasets (GSE138206, GSE13601, and GSE78060) were retrieved using (www.ncbi.nlm.nih.gov/geo). The GSE138206 dataset was submitted by Pan et al. (Estilo et al., 2009) and was constructed using the Affymetrix Human Genome U133 Plus 2.0 Array GPL570 [HG-U133 Plus 2]. The GSE138206 dataset contains 18 samples, comprising six tongues squamous cell carcinoma (TSCC) tissues, 12 adjacent to cancer, and contralateral normal tissues. Singh et al. (Estilo et al., 2009) used the GSE13601 dataset, which utilized GPL8300 [HG_U95Av2]. There were 58 samples in the GSE13601 collection, including 31 TSCC and 27 normal tissues. Enokida et al. submitted GSE78060 that utilized GPL570 [HG-U133_Plus_2]. There were 30 samples in the GSE78060 dataset, with 26 TSCC and four normal tissue margins.

### 2.2 Differential expression analysis

Furthermore, the GSE78060 dataset contains only four normal samples, and using a small number of samples may hinder statistical analysis and result in inaccurate conclusions. We performed a differential analysis (|Log2 (fold change)| (|Log2FC|) > 2 and adjusted *p*-value < 0.05) in the R computer environment by comparing tumor tissues to normal tissues. To avoid obtaining less accurate results, we utilized Venny (bioinfogp.cnb.csic.es/tools/venny/) to build a Venn layout from down/upregulated gene intersections in tumor tissues relative to non-tumor tissues in the three datasets.

### 2.3 Gene expression in human cancers

Tumor Immune Estimation Resource, (TIMER2.0) (timer.comp-genomics.org/) (Li et al., 2020).

We inserted *CLU* onto TCGA project “Gene DE” module of the “Exploration” part and then investigated the changes in *CLU* expression among neighboring non-tumor tissues and various cancers or distinct subtypes.

Gene Expression Profiling Interactive Analysis Version 2 (GEPIA2) (gepia2.cancer-pku.cn/#analysis) (Tang et al., 2019).

Some cancers in TCGA project that lack or have very minimal healthy tissues unrelated to non-tumor tissues, including TCGA-ACC (adrenocortical carcinoma) and TCGA- MESO (mesothelioma). Moreover, we utilized “Expression Analysis-expression DIY-Box Plots” module to depict the expression variances between tumor tissues and non-tumor ones. (Cutoff value settings: |Log2FC| = 1, *p*-value = 0.01, and “Match TCGA normal and GTEx data”).

UALCAN (web: ualcan.path.uab.edu/index.html) (Chandrashekar et al., 2017).

The Clinical Proteomic Tumor Analysis Consortium (CPTAC) database was used to compare the total protein expression levels across six varieties of tumors and neighboring non-tumor tissues. The selected tumor datasets included breast cancer (BC), ovarian cancer, colon cancer (CC), lung adenocarcinoma (LUAD), uterine corpus endometrial carcinoma (UCEC), and HNSC. By putting “*CLU*” into the TCGA database, we could compare the methylation levels of the primary tumor and non-tumor tissues.

### 2.4 Pan-cancer survival prognosis analysis

We utilized GEPIA2’s “Survival Map” and “Survival Analysis” module to acquire Overall survival (OS) and Disease-free survival (DFS) significance data for *CLU* across all TCGA tumors (settings: cutoff-high value:50%, cutoff-low value:50%). Log-rank tests were used to test the hypotheses. Survival plots were used to analyze the data.

### 2.5 Gene mutation analysis in human cancers

cBioPortal (web: www.cbioportal.org/) (Gao et al., 2013).

In the section “Quick select,” we entered “TCGA Pan-Cancer Atlas Studies” then typed “*CLU*” to obtain the genetic mutation features of *CLU*. The “Cancer Types Summary” module displayed Copy number alteration (CNA) frequency and type findings across all TCGA tumors. The “Mutations” module may display *CLU*’s mutated site information. We also utilized the “Comparison/Survival” module to acquire data on the differences in OS, progression-free survival (PFS), and DFS between TCGA tumor patients with and without *CLU* genetic mutation. The data are demonstrated using Kaplan-Meier plots.

### 2.6 Analyses of immune infiltration

The web-based “Immune-Gene” TIMER2 module was used to examine the correlation between *CLU* expression, and immunological infiltrates in all TCGA tumors. Cancer-associated fibroblasts (CAFs) CD8^+^ and T cells are used as immunological cells. Immunological infiltrates were assessed using the TIMER, CIBERSORT, CIBERSORT-ABS, QUANTISEQ, XCELL, MCPCOUNTER, and EPIC algorithms. *p*-values and partial correlation (cor) values were calculated using the purity-adj-Spearman’s correlation test. The data are represented graphically by a scatter plot along with heatmaps.

### 2.7 Analysis of gene set enrichment

STRING (web: string-db.org/) (Szklarczyk et al., 2021).

We began with (“*Homo sapiens*”) and a single (“*CLU*”). After that, we decided on some critical variables, such as the minimum acceptable interaction score ["Low confidence (0.150),” the significance of network edges (“evidence”), the maximum interactors number to demonstrate (“no more than fifty interactors” in the first shell), and the sources of interactions themselves (which were set to “active”) (“experiments”). All *CLU*-bound proteins identified in the experiments were acquired.

Using data from all TCGA tumor and non-tumor tissues, we applied “Similar Gene Detection” module of GEPIA2 to determine the top 100 *CLU*-correlated directed genes. Additionally, we analyzed Pearson correlation of *CLU* and specific genes using the “correlation analysis” module in GEPIA2. We used a dot plot to illustrate data for the four highest correlation coefficients. Furthermore, the “Gene Corr “module in the " Explore “section was used to generate the selected gene heatmaps. The adj-Spearman’s correlation analyses were utilized for (cor) and *p*-values calculations.

Metascape (web: metascape.org/gp/index.html#/main/step1) (Zhou et al., 2019).

We uploaded the gene lists to metascape, chose “paste a gene list,” pasted in the *CLU*-binding and interacting genes, and submitted the annotation chart data. “Pathway & Process Enrichment” and “Protein-protein Interaction Enrichment” are among the outputs of the Enrichment analysis. MCODE is used to demonstrate the protein networks built based on the physical connections between the input protein candidate lists.

In addition, we utilized “ClusterProfiler” module for Kyoto Encyclopedia of Genes and Genomes (KEGG) along with gene ontology (GO) enrichment studies. “ggplot2″ and “enrichplot” R tools were used to illustrate the enrichment analysis findings. This research made use of R programming [R-4.1.2] (www.r-project.org/).

### 2.8 Statistical analysis

Wilcoxon rank sum test and Kruskal–Wallis test were used to evaluate *CLU* expression levels between the two groups. The hazard ratio (HR) and *p*-value for survival analysis were computed using a univariate Cox regression analysis. Kaplan-Meier analyses were used to assess OS in patients with high or low *CLU* expression. Statistical significance was set at *p* < 0.05.

## 3 Results

### 3.1 GEO data analysis

GEO database was used to gather gene expression data for 60 TSCC and 112 normal tissues. The number (DEGs) number found in each of the three datasets is illustrated in Supplementary Table S1.

**FIGURE 1:**
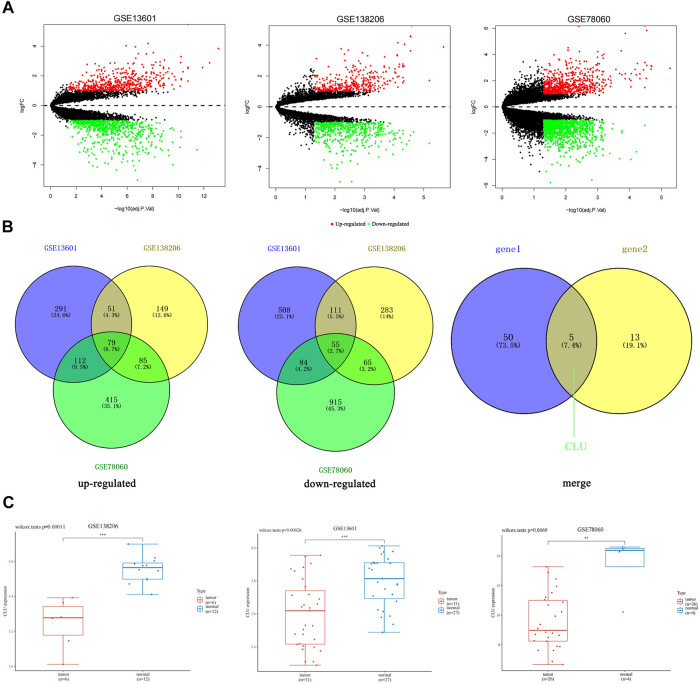
Differentiation of gene expression signatures among TSCC datasets from GEO database. **(A)** The number of DEGs found from the three GEO datasets was shown by volcano plots; **(B)** Venn diagram demonstrates the intersections of genes among the three GEO datasets; additionally, the intersection between this intersection and the down-regulated genes in the whole gene transcriptome data; **(C)** the box plot illustrated the expression distribution of *CLU* in tissues, where the upper left corner displays the *p*-value for significance.

The volcano plots in Figure 1A displayed the DEGs number reported from each of the three datasets. In GSE138206, we found 1756 DEGs, of which 972 were upregulated, and 784 were downregulated. In GSE13601, we found 4,230 DEGs, of which 1,437 were upregulated, and 2,793 were downregulated. In addition, we discovered 1810 DEGs in GSE78060, with 691 being upregulated and 1,119 being downregulated. The Venn diagram depicts the gene crossovers among the three GEO datasets. (Figure 1B). In tumor tissues, 79 genes were upregulated relative to normal tissues, whereas 55 genes were downregulated. Furthermore, we intersected the 55 downregulated genes obtained with the downregulated genes in the TSCC whole gene transcriptome data, as illustrated in Figure 1B, gene1 represents the 55 genes obtained by intersecting the downregulated genes from each of the three data sets, and gene2 represents the downregulated genes in the whole gene transcriptome data. Five genes, including *CLU,* were identified (Supplementary Table S2 **)**.

To evaluate DEGs with statistical significance, we considered GSE78060 as an example, |Log2FC| > 3, and *p* < 0.05 as the screening criteria. Three genes, *CLU*, *ADH1B*, and *CRISP3,* were screened. Despite the fact that *CRISP3* expression has been linked to the genesis and progression of prostate and breast malignancies (Wang et al., 2019; Volpert et al., 2020), *CRISP3* exhibited non-significant expression in pan-cancer tissues (Supplementary Figure S1), and *ADH1B* has been verified in cancer (Li et al., 2017); therefore, *CLU* was selected.

Then, we utilized the “Home for Researchers” (web: www.aclbi.com/static/index.html#/geo) to evaluate expression levels of single genes in several groups of samples in three datasets and discovered that *CLU* was significantly down-regulated in tumors relative to non-tumor tissues. (*p* < 0.01, Figure 1C).

### 3.2 Gene expression analysis

Our study aimed to examine the carcinogenic properties of human *CLU*. We examined *CLU* expression in various human tissues. As demonstrated in Supplementary Figure S2, the highest *CLU* protein expression was found in the Bronchus, Epididymis, Endometrium, Cervix, and Tonsil. In terms of RNA tissue specificity, *CLU* was demonstrated to be significantly expressed in the medulla oblongata, hypothalamus, and liver in HPA datasets (web: www.proteinatlas.org). *CLU* was significantly expressed in the spinal cord, liver, pons, and medulla in functional annotation of the mammalian genome (FANTOM5) datasets (web: fantom.gsc.riken.jp/5/) and significantly expressed in the liver, adrenal gland, and retina in the GTEx datasets (web: gtexportal.org/home/).

**FIGURE 2:**
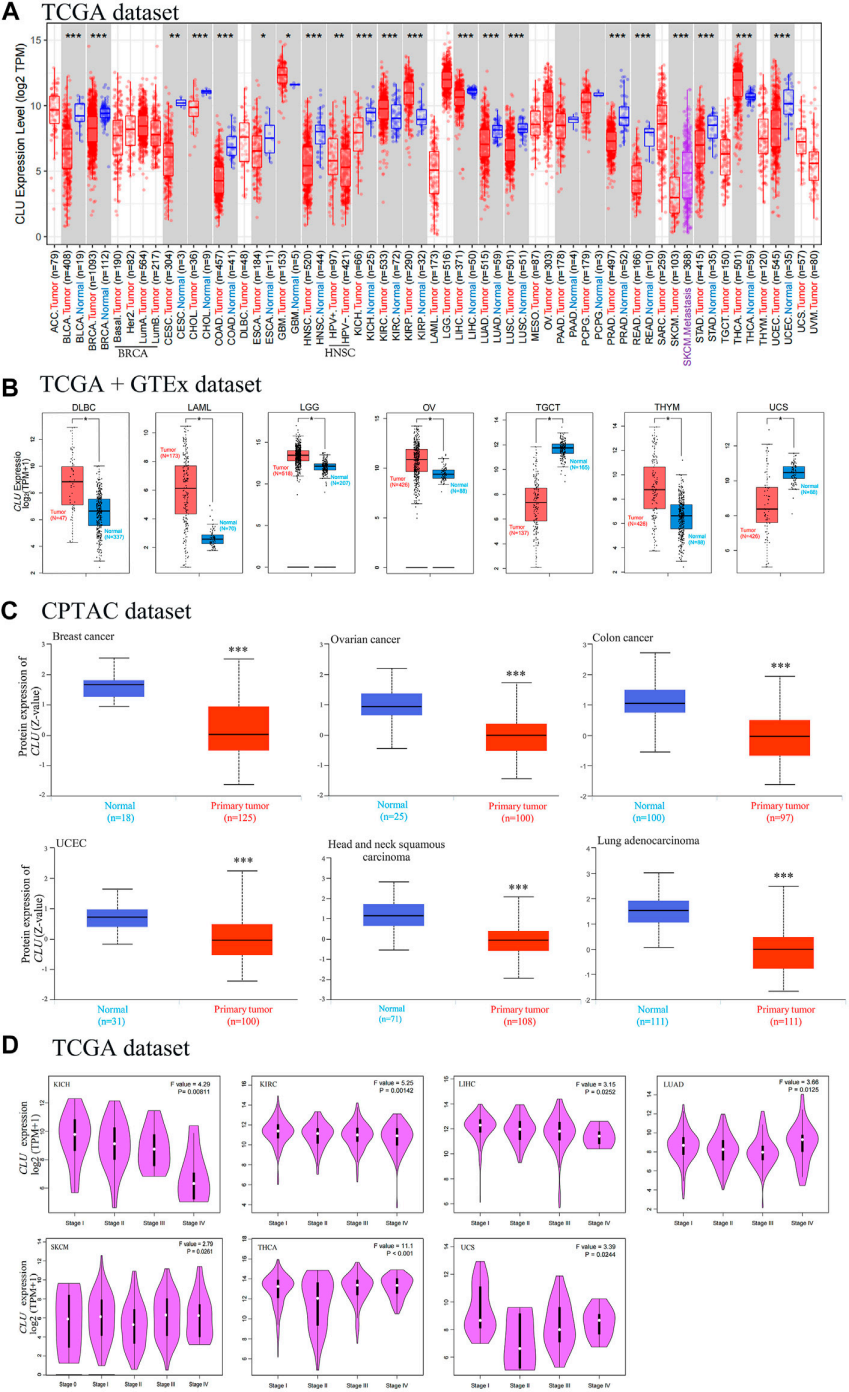
Degree of *CLU* expression in various tumor tissues and stages. **(A)** TIMER2 was used to assess the TCGA project’s *CLU* gene expression differences in distinct cancers or particular tumor subtype tissues and neighboring non-tumor tissues (*p < 0.05; **p < 0.01; ***p < 0.001); **(B)** the matching non-tumor tissues were used as controls in the GTEx database, and GEPIA2 was utilized to examine *CLU* expression status among DLBC, LAML, LGG, OV, TGCT, THYM, and UCS cancers (*p < 0.05); **(C)** the CPTAC was utilized to compare *CLU* total protein expression in non-tumor and tumor tissues from BC, CC, ovarian cancer, UCEC, HNSC, and LUAD (***p < 0.001); **(D)** through utilizing TCGA dataset, GEPIA2 was used for *CLU* expression gene analysis at various pathological staging (stage I to V) in KICH, KIRC, LIHC, LUAD, SKCM, THCA, and UCS cancers.

The TIMER2 method was utilized to analyze *CLU* expression status across diverse tumor types in TCGA. As displayed in Figure 2A, *CLU* expression in tumor tissues of kidney chromophobe (KICH), prostate adenocarcinoma (PRAD), breast invasive carcinoma (BRCA), cholangiocarcinoma (CHOL), bladder urothelial carcinoma (BLCA), liver hepatocellular carcinoma (LIHC), LUAD, LUSC, UCEC, colon adenocarcinoma (COAD), rectum adenocarcinoma (READ), skin cutaneous melanoma (SKCM), HNSC, stomach adenocarcinoma (STAD) (*p <* 0.001), cervical squamous cell carcinoma and endocervical adenocarcinoma (CESC) (*p <* 0.01), and esophageal carcinoma (ESCA) (*p <* 0.05) was lower than that in the corresponding non-tumor tissues. The prevalence of kidney renal papillary cell carcinoma (KIRP), kidney renal clear cell carcinoma (KIRC), thyroid carcinoma (THCA) (*p <* 0.001), HNSC (HPV+/HPV-) (*p <* 0.01), and glioblastoma multiforme (GBM) (*p <* 0.05) was greater than that of the comparable healthy tissues.

We further examined *CLU* expression differences among non-tumor tissues and tumor tissues of Uterine Carcinosarcoma (UCS), Acute Myeloid Leukemia (LAML), lymphoid neoplasm diffuse large B-cell lymphoma (DLBC), testicular germ cell tumors (TGCT), ovarian serous cystadenocarcinoma (OV), low-grade brain glioma (LGG), and thymoma (THYM) (Figure 2B, *p <* 0.05). As depicted in Supplementary Figure S3, no statistical changes were observed for the other types, including ACC and Sarcoma (SARC).

According to the findings of the CPTAC project, total *CLU* protein expression was lower in primary tissues of BC, HNSC, CC, UCEC, LUAD, and ovarian cancer (Figure 1C, *p <* 0.001) than in non-tumor tissues.

By utilizing GEPIA2’s module “Pathological Stage Plot” to evaluate the relationship between *CLU* expression and the pathological staging of malignancies such as KICH, KIRC, LIHC, LUAD, Skin Cutaneous Melanoma (SKCM), THCA, and UCS (Figure 2D, all *p* < 0.05), excluding others (Supplementary Figure S3).

### 3.3 Survival data analysis

**FIGURE 3:**
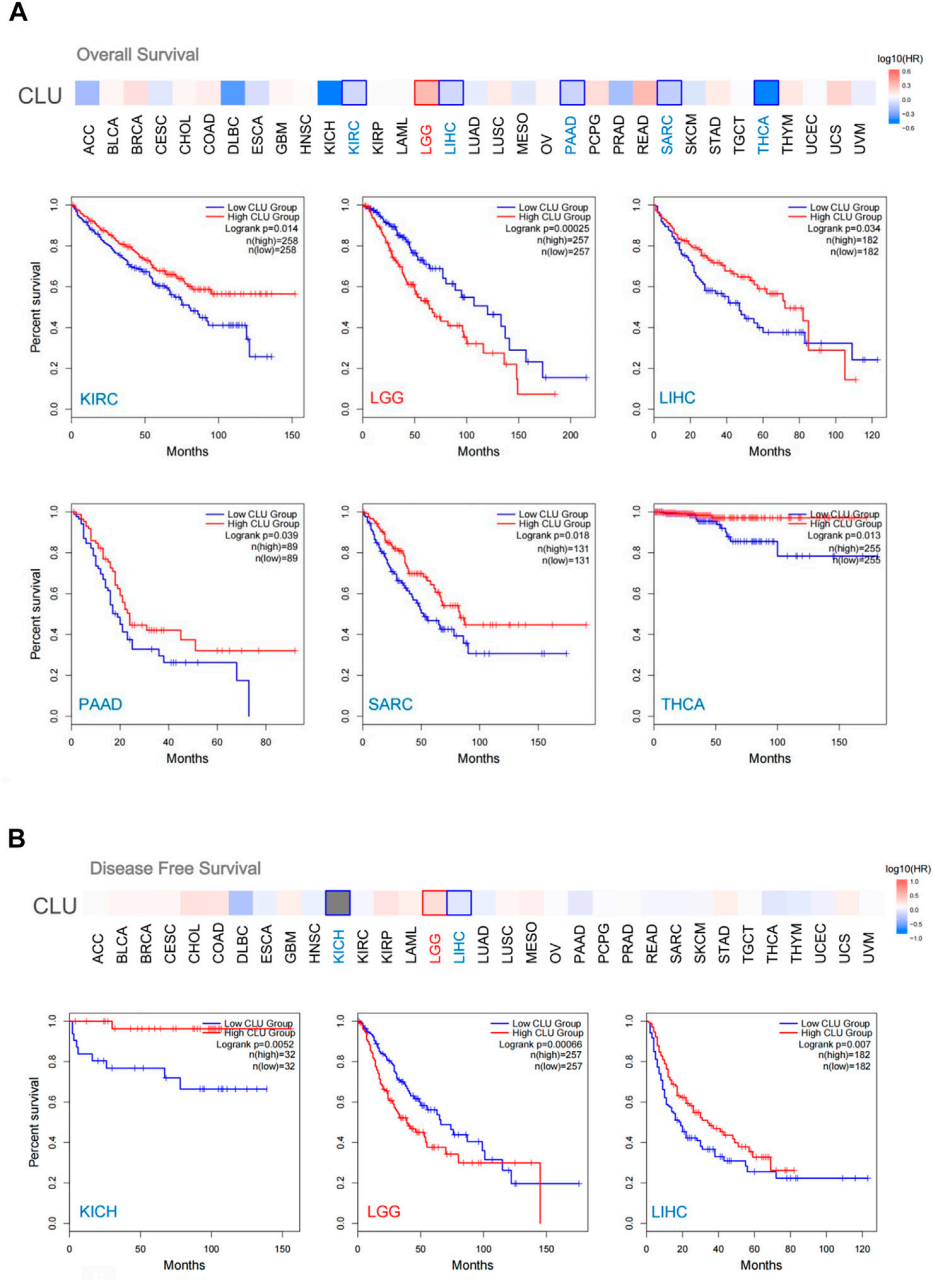
GEPIA2 was utilized to investigate the relation between CLU gene expression and OS prognosis in all TCGA cancers. **(A)** OS analysis; **(B)** DFS analysis, survival map and Kaplan-Meier plots provided encouraging findings with substantial differences.

Cancer cases are categorized into two groups based on *CLU* expression levels. We analyzed the link between patient prognosis and cancer types using the TCGA and GEO datasets. Low expression of *CLU* was correlated with worse OS prognosis for KIRC patients (*p =* 0.014), LIHC (*p =* 0.034), PAAD (*p =* 0.039), SARC (*p =* 0.018), and THCA (*p =* 0.013) in TCGA, as illustrated in Figure 3A **.** Data from DFS analyses (Figure 3B) revealed a link between *CLU* low expression and worse prognosis in TCGA for KICH cases (*p =* 0.0052) and LIHC (*p =* 0.007). Furthermore, a worse OS prognosis is linked to higher *CLU* gene expression in LGG (Figure 3A, *p <* 0.001) and poor DFS prognosis among LGG (Figure 3B, *p <* 0.001).

In addition, the Kaplan-Meier plotter utility examined OS data and the prognosis of various tumor patients. Low *CLU* expression was associated with poor OS (*p <* 0.001), first progression (FP) (*p <* 0.001), and post-progression survival (PPS) (*p* = 0.029) in patients with LAUD (Supplementary Figure S4 **)**. Furthermore, decreased *CLU* expression correlated with poor OS (*p <* 0.001), PFS (*p <* 0.001), and relapse-free survival (RFS) (*p =* 0.0011) in patients with hepatic cancer (Supplementary Figure S4). However, in gastric cancer patients, increased *CLU* expression was associated with poorer OS (*p <* 0.001), FP (*p =* 0.013), and PPS (*p <* 0.001) (Supplementary Figure S4). These findings suggest that *CLU* expression is linked to pan-cancer patient prognosis; however, various cancer patients have distinct prognoses.

### 3.4 Genetic alteration analysis

**FIGURE 4:**
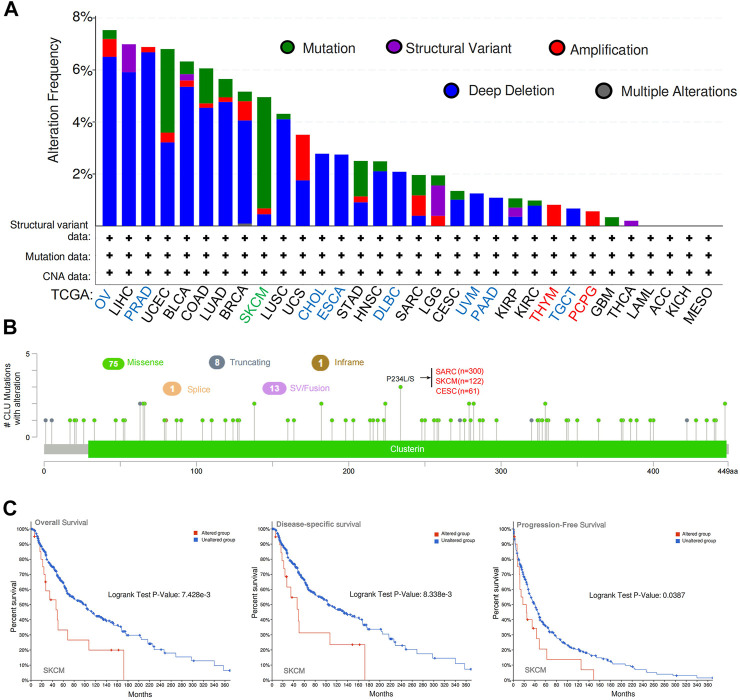
cBioPortal was utilized to examine CLU gene’s mutation features and prognostic value in several TCGA cancers. **(A)** mutation type and frequency of modification in diverse cancers; **(B)** *CLU* mutation location; **(C)** probable correlation between *CLU* mutation status and SKCM OS, disease-specific, and PFS prognoses.

*CLU* genetic modification was examined in various tumor tissues from TCGA cohort. As demonstrated in Figure 4A, OV patients with “Deep Deletion” as the major type had the greatest *CLU* modification frequency (>8%). In PRAD cases (6%**–**8% genetic alteration frequency), the “Deep Deletion” type of *CLU* was the prevalent variety, followed by LIHC, BLCA, COAD, LUAD, BRCA, and LUSC cases (4%**–**6% genetic alteration frequency). The “Mutation” kind of *CLU* was the most common in the SKCM patients, which exhibited a frequency variation of ∼5% (Figure 4A). Notably, every CHOL, ESCA, DLBC (2%**–**4% frequency), UVM, PAAD, and TGCT (∼2% frequency) patient with genetic mutations showed *CLU* copy number deletion. (Figure 4A, B) displays the types, locations, and numbers of *CLU* genetic mutations. We discovered that the most common genetic alteration was *CLU* gene missense mutation and P234 L/S modification in the clusterin domain, which was identified among 300 SARC patients, 122 cases of SKCM, and 61 cases of CESC (Figure 4B), and is capable of inducing a missense mutation of the *CLU* gene, translation from P (proline) to L (leucine) at the 234 sites of *CLU* protein. In addition, we investigated whether a correlation exists between a mutation in the *CLU* gene and a worse prognosis for patients’ clinical survival in several cancer types. As illustrated in Figure 4C, SKCM cases with changed *CLU* had a superior overall prognosis (*p* < 0.001), disease specificity (*p <* 0.001), and PFS (*p =* 0.0387), excluding DFS, relative to patients without *CLU* mutations.

These findings suggest that *CLU* expression in pan-cancer is associated with *CLU* mutation and copy deletion number and that *CLU* genetic modification is directly associated with the OS prognosis of diverse tumor patients.

### 3.5 DNA methylation analysis

**FIGURE 5:**
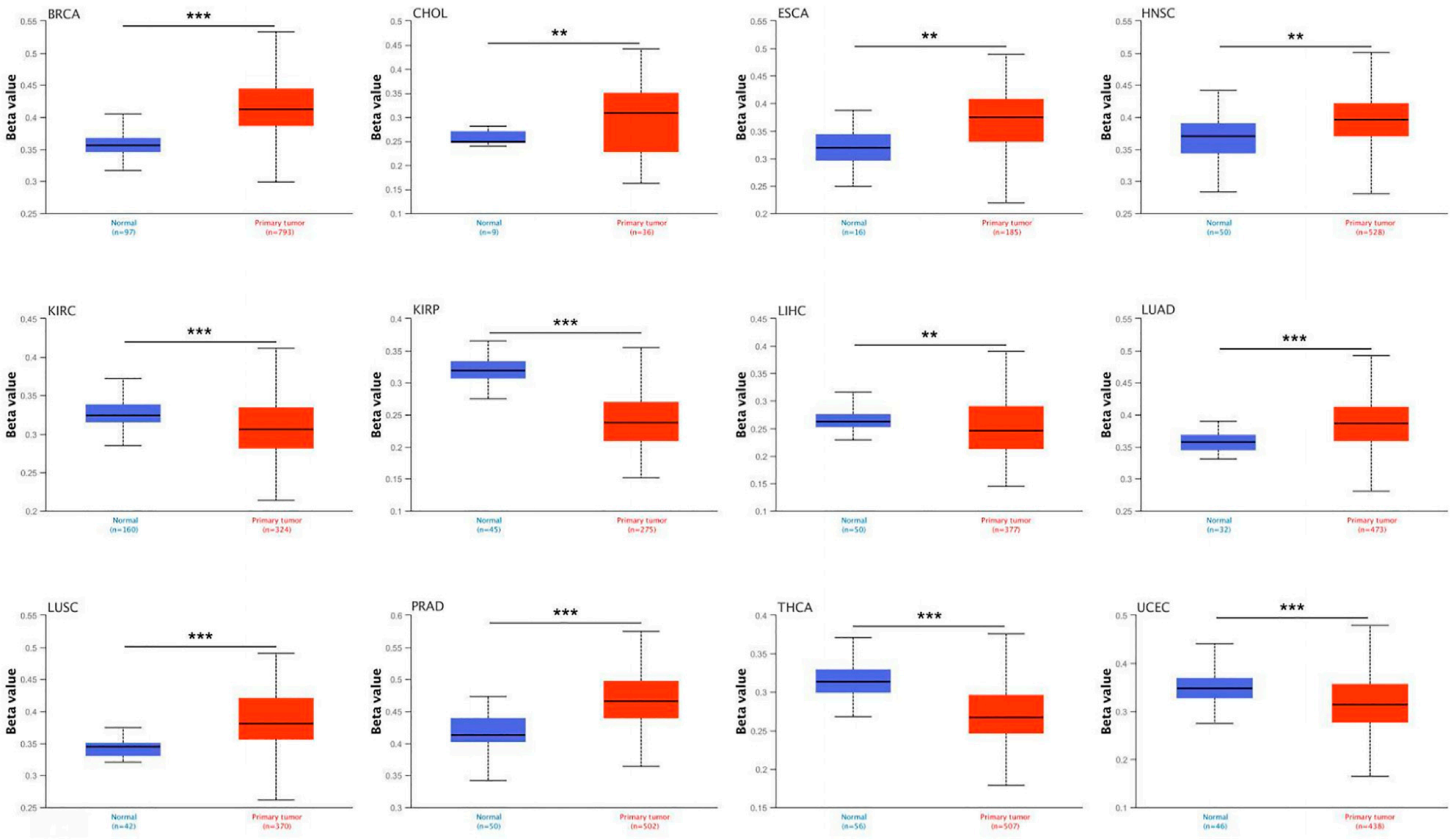
*CLU* promoter methylation in pan-cancer. Data came from UALCAN datasets.

Both cancer incidence and progression are influenced by DNA methylation. We studied *CLU* DNA methylation using UALCAN and TCGA datasets. A total of 23 different types of malignancies were studied (SARC, LUAD, CHOL, STAD, KIRC, THCA, COAD, KIRP, LIHC, LUSC, PAAD, UCEC, ESCA BLCA, HNSC, PCPG, BRCA, GBM, TCGT, PRAD, READ, THYM, and CESC). According to the UALCAN database, a considerable increase in methylation levels was found across *CLU* in LUAD, CHOL, HNSC, ESCA, BRCA, PRAD, and LUSC tissues compared to that in non-tumor tissues (Figure 5). *CLU* methylation was considerably reduced in KIRC, KIRP, LIHC, THCA, and UCEC cells (Figure 5). This finding warrants further in-depth research.

### 3.6 Immune infiltration analysis

**FIGURE 6:**
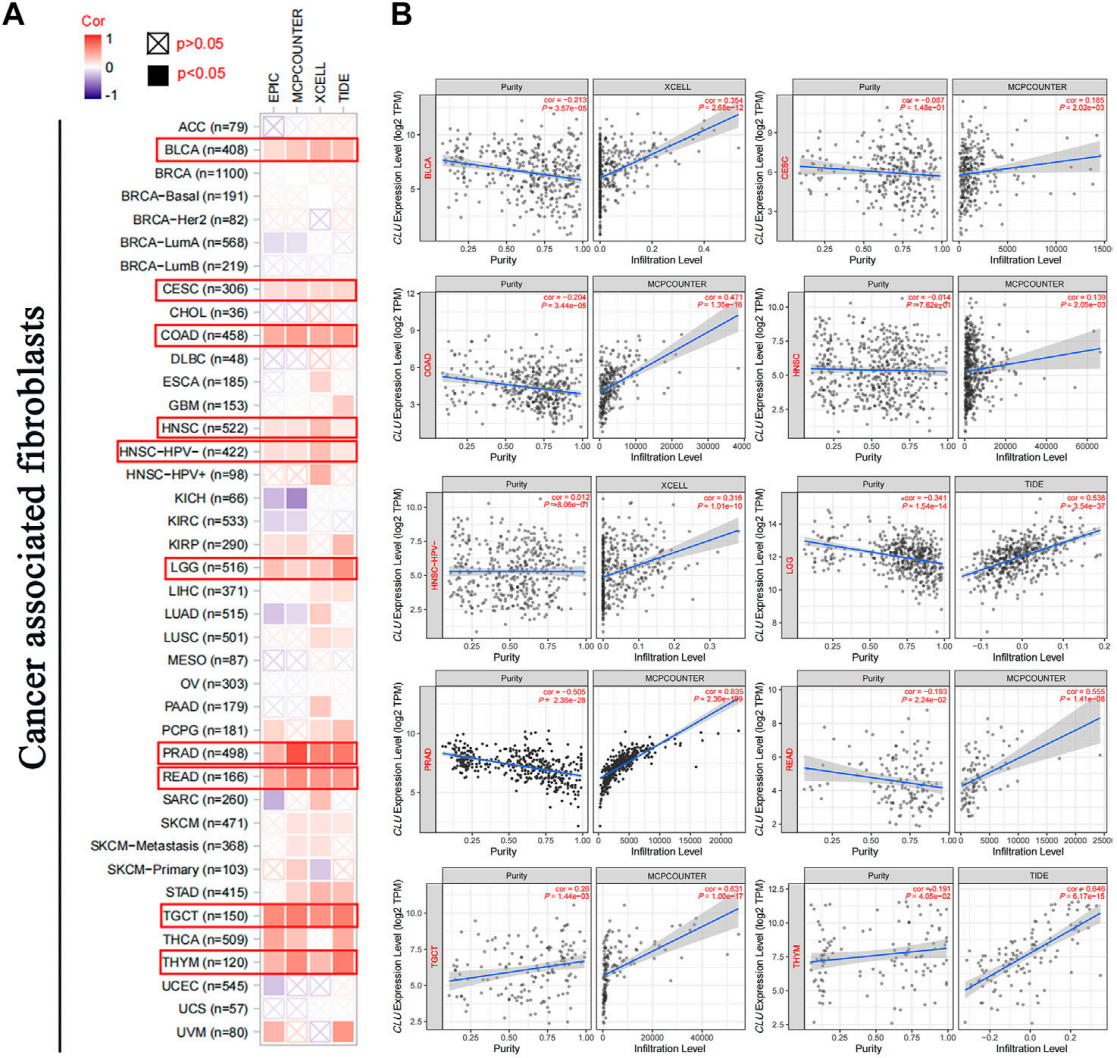
Correlation investigation of CLU gene expression and CAFs immune infiltration. **(A)** For all TCGA tumors, different algorithms (MCPCOUNTER, EPIC, XCELL, and TIDE) were used to assess the connection between *CLU* expression and the amount of immunological infiltration of CAFs; **(B)** scatterplot of the chosen tumor was provided, which was created using one of the methods.

Tumor-infiltrating immunological cells are involved in cancer initiation, development, and metastasis. Several tumor-infiltrating immunological cell functions have been modulated by CAFs in the stromata of the tumor microenvironment. We investigated the possibility of a correlation between various TCGA CAFs infiltration types and *CLU* gene expression (Figure 6). We discovered that CAFs estimated infiltration among TCGA cancers CESC, PRAD, COAD, HNSC, TGCT, HNSC-HPV-, LGG, BLCA, READ, and YHYM examined using all methods was statistically favorably connected to *CLU* expression. Furthermore, we discovered that the predictive infiltration of CD8^+^ T-cell immune infiltration was significantly associated with *CLU* expression in PAAD and STAD cancers but inversely correlated with KIRC and THYM tumors (Supplementary Figure S5). Scatterplots for the previously stated tumor developed using one of the methods are displayed in Supplementary Figure S6; Supplementary Figure S5. According to the A General Toolbox for Identifying Object Detection Errors (TIDE) algorithm, *CLU* expression in LGG was positively correlated with the amount of CAFs infiltration (cor = 0.538, *p <* 0.001) (Figure 6B).

### 3.7 CLU-related gene enrichment analysis

**FIGURE 7:**
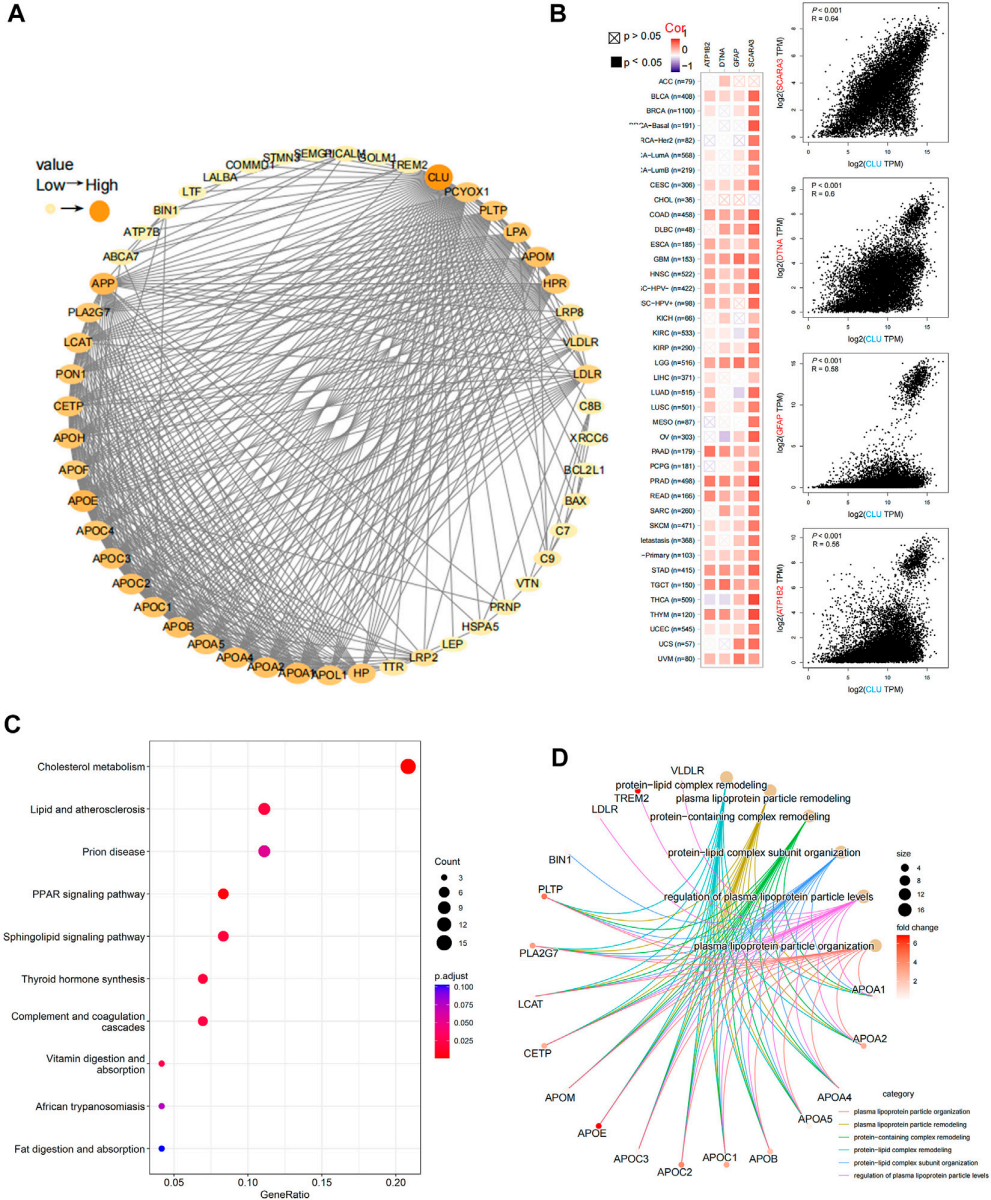
Analysis of CLU-related gene enrichment. **(A)** The top 50 *CLU*-bound proteins were identified experimentally using STRING program and depicted using a molecular interaction network; **(B)** utilizing GEPIA2 tool, the top 100 *CLU*-related genes in TCGA projects were collected, and the expression correlation among *CLU* and the top four targeted genes (SCARA3, DTNA, GFAP, and APT1B2) was examined, the matching heatmap data for the cancer types selected are presented; **(C)** analysis of KEGG pathways related *to CLU*-bound and interacting genes; **(D)** Gene Ontology (GO) study of *CLU*-bound and interacting genes.

Using STRING, 50 experimentally validated *CLU*-bound proteins were identified. The protein network interactions are depicted in Figure 7A. Furthermore, GEPIA2 is used to merge TCGA projects of overall tumors and neighboring non-tumor tissues to obtain 100 leading targeted genes significantly associated with *CLU* expression. Figure 7B displays that *CLU* expression was significantly associated with ATP1B2 expression (ATPase Na+/K+ transporting subunit beta 2), DTNA (dystrobrevin alpha) (R = 0.6), GFAP (glial fibrillary acidic protein) (R = 0.58), and SCARA3 (scavenger receptor class A member 3) (R = 0.64) genes (all *p <* 0.001). Most cancer types exhibited a strong correlation between these four genes and *CLU* expression levels (Figure 7B). Furthermore, these two databases were used to perform KEGG pathway and GO enrichment analyses. The findings show that “cholesterol metabolism,” “lipid and atherosclerosis,” and “PPRA signaling pathway” PPRA signaling pathway may play a role in *CLU*’s impact of *CLU* on carcinogenesis and proliferation. According to the GO enrichment analysis results, the bulk of these genes are involved in DNA metabolism or cellular process pathways, including cholesterol transport, protein-lipid complexes, phospholipid binding, plasma lipoprotein particle remodeling, sterol transport, and plasma lipoprotein particle level regulation (Figure 7D, and Supplementary Figure S6).

## 4 Discussion

OSCC is common cancer that develops in oral epithelium. The most frequent type of OSCC, TSCC, is characterized by aggressive biological activity and poor survival (Sano and Myers, 2007; Karatas et al., 2017). Recent studies have suggested a significant interaction between *CLU* and OSCC (Kadam et al., 2021). This study aimed to examine the function of *CLU* gene, which was retrieved from the tongue squamous carcinoma dataset, in OSCC, HNSC, and diverse human cancers. This study is the first to evaluate *CLU* expression in a pan-cancer dataset thoroughly. Compared to paracancerous and non-tumor tissues, BLCA, UCS, BRCA, CHOL, LUAD, COAD, ESCA, STAD HNSC, KICH, LIHC, LUSC, PRAD, CESC, READ, SKCM, TGCT, and UCEC demonstrated a significant decrease in *CLU* expression. (Figures 2A, B). In various malignancies, lower *CLU* expression is linked to poorer OS, DFS, FP, PPS, and PFS (Supplementary Figure S3; Supplementary Figure S4). *CLU* gene deletions were found in virtually all malignancies and were associated with the prognosis of most cancer patients*.* (Figure 4). *CLU* expression was significantly associated with immunological infiltration and checkpoint markers in several cancers (Figure 6). Our GO and KEGG analyses indicated that *CLU* was significantly associated with many signaling pathways, which is consistent with earlier research (Figure 7). In conclusion, our research contributes to the development of *CLU*-targeting therapy options by highlighting *CLU* utilization as a possible predictive biomarker in immuno-oncology for several malignancies.

Clusterin *(CLU)* is an ATP-independent, stress-activated molecular chaperone typically produced by cells (Wilson and Zoubeidi, 2017). *CLU* has been associated with carcinogenesis and tumor growth in various cell types. Human cells produce two distinct *CLU* isoforms. A nuclear version of *CLU* protein (n*CLU*) promotes apoptosis, but the secretory form (s*CLU*) promotes survival (Shannan et al., 2006). The incidence and progression of multiple cancers, such as lung cancer, prostate cancer, BC, CC, and HCC, have been linked to *CLU*. (Mazzarelli et al., 2009; Panico et al., 2009; Redondo et al., 2009; Rizzi and Bettuzzi, 2009; Patarat et al., 2021). However, it is uncertain whether *CLU* influences carcinogenesis, development, and metastasis *via* common molecular processes. Accordingly, we used the existing TGCT, GEO, and CPTAC datasets to examine the link between *CLU* and different malignancies according to their prognosis and molecular processes.

First, we checked the changes in *CLU* expression among tumor tissues and non-tumor tissues that matched the tumor tissues. The results indicated that *CLU* expression levels differed significantly among the 21 types of tumor tissues and non-tumor tissues that matched the tumor tissues. We conclude that *CLU* is expressed lower in most cancers than non-tumor tissues. *CLU* has been reported to have tumor suppressor activity in prostate, lung, and oral cancers, and low expression of *CLU* is associated with worse prognosis and genetic instability (Panico et al., 2009; Rizzi and Bettuzzi, 2009; Kadam et al., 2021). This finding is in agreement with the results of the present study.

Herein, GEPIA2 was used to find a correlation between *CLU* expression and tumor prognosis in the TCGA database. We discovered that lower *CLU* expression was correlated with worse OS prognosis for malignancies of KIRC, LIHC, PAAD, SARC, and THCA, excluding LGG. *CLU* expression has also been associated with worse survival in cancer patients in many recent studies, including KIRC and LIHC (Liu et al., 2018; Zheng et al., 2020). However, higher expression of *CLU* in tumor tissues leads to a worse prognosis of cancer in these studies, and we believe that this *CLU* gene is s*CLU*, which has a negative effect on cancer prognosis. The mechanism may be that s*CLU* affects apoptosis by regulating different signaling pathways in a variety of tumours and interacting with signal transduction proteins. For example, s*CLU* inhibits mitochondrial apoptosis in hepatocellular carcinoma by suppressing the expression of Gadd45a, a negative regulator of pro-apoptotic properties of AKT, activating the PI3K/AKT axis and subsequently upregulating the expression of the apoptotic protein B-cell lymphoma-2 (Bcl-2) (Wang et al., 2018). s*CLU* has the potential as a biomarker in the diagnosis and prognosis of several malignancies, including liver cancer, osteosarcoma, and BC (Yao et al., 2018; Ma et al., 2019; Chen et al., 2021).

Furthermore, the question of whether anti-apoptotic s*CLU* is the sole type of *CLU* expressed in cancer or if n*CLU* is downregulated in different tumor entities remains unanswered. We suppose that improper *CLU* expression is strongly associated with a worse survival prognosis for most malignancies. In contrast, the OS prognosis study data for *CLU* gene exhibited varied conclusions in numerous tumors.

*CLU* gene expression has been linked to an elevated risk of lung cancer (Chen et al., 2020; Tan et al., 2021; Yuan et al., 2021). Nevertheless, in the TCGA-LUAD/LUSC cohort, we could not examine the correlation between *CLU* expression and the survival prognosis of lung cancer patients, which might be due to various data processing or revised survival information. Kaplan-Meier plots of survival data generated with Affymetrix 222,043 microarrays revealed that decreased *CLU* expression was linked to worse OS, FP, and PPS prognoses in patients with lung cancer. Furthermore, we discovered an association between low *CLU* expression and worse OS, PFS, and RFS in patients with hepatic tumors, which agrees with our results from the GEPIA2 algorithm in TCGA database. *CLU* has a tumor-suppressing effect on lung cancer (Chen et al., 2020); similarly, our findings suggest that decreased *CLU* gene expression may contribute to a worse prognosis in lung cancer patients. However, increased expression of *CLU* in tumor tissues has been linked to worse prognosis in several investigations of liver cancer (Zheng et al., 2020). Consequently, we feel that further in-depth molecular experimental data are still required to assess whether *CLU* expression is important in the tumor start mechanism indicated above.

Using TCGA data, we first investigated *CLU* molecular mechanisms in different cancers based on *CLU* DNA methylation. Hypermethylated *CLU* expression significantly decreases in untreated and hormone-resistant human prostate cancer (Rauhala et al., 2008). Our research also depicted a significant increase in the methylation level of *CLU* in PRAD tissues compared to that in non-tumor tissues. Although promoter hypermethylation can inhibit the expression of *CLU* and demethylated cells can promote apoptotic cell death by inducing n*CLU* (Lund et al., 2006; Park et al., 2014), no research has been conducted on the possible function of *CLU* methylation in more tumors. Hence, this may require further evaluation of the possible significance of *CLU* methylation in the initiation and advancement of various tumors.

The surrounding tumor microenvironment (TME) affects cancer cell survival, proliferation, migration, and even dormancy. CAFs have multiple functions in tumor formation inside the TME (Biffi and Tuveson, 2021). CAFs enhance cancer growth *via* pleiotropic processes (Liao et al., 2019; Nurmik et al., 2020); However, in some cases can inhibit tumor progression (Miyai et al., 2020). Several approaches have been used to determine the association between *CLU* levels and tumor-related immunological cells. Herein, immunological infiltration of distinct tumor-related immunological cells was associated with *CLU* expression in CAFs and CD8 + T cells. *CLU* expression positively correlates with immune cell infiltration in most cancers, including BLCA, CESC, HNSC, LGG, and PRAD. *CLU* was found to be expressed at a lower level in the high-risk C3 subtype of prostate cancer, with significantly less infiltration of CD8 T cells, monocytes, resting dendritic cells, activated dendritic cells, and activated mast cells, implying that *CLU* may influence immune cell infiltration through some mechanism (Zhang et al., 2020). We inferred that *CLU* might affect patient survival by altering immune cell infiltration in the TME; however, further studies are needed.

*CLU*-bound protein and *CLU* expression-related gene enrichment investigations across all cancer types revealed the possible relevance of “cholesterol metabolism,” “lipid and atherosclerosis,” “phospholipid binding,” and “protein-lipid complex” in cancer’s etiology or pathogenesis. *CLU* functions as a molecular chaperone in various physiological processes that contribute to carcinogenesis and tumor formation, including apoptotic cell death, cell cycle control, DNA repair, cell adhesion, tissue remodeling, and lipid transport (Pucci et al., 2009; Praharaj et al., 2021; Uddin et al., 2021). It was discovered that by activating the PI3K/AKT axis, s*CLU* could significantly promote the transcription of matrix metalloproteinase-2 (MMP-2), a protein related to lipid metabolism, and inhibit the expression of E-calmodulin, thereby inducing tumor invasion in hepatocellular carcinoma, suggesting that *CLU* may influence tumour invasion and migration by affecting the pathway of lipid transport (Zhong et al., 2018). According to several studies, s*CLU* works as a chaperone for misfolded proteins and is expected to support survival by lowering oxidative stress; n*CLU* functions as a prodeath signal, preventing cell proliferation and survival (Shannan et al., 2006; Bettuzzi and Rizzi, 2009). These findings might aid in deciphering the role of *CLU* in the etiology of various cancers.

## 5 Conclusion

In conclusion, our pan-cancer study demonstrated that *CLU* expression is low in most malignancies and significantly correlates with clinical prognosis, DNA methylation, and immunological cell infiltration in cancer patients. These results provide a thorough understanding of *CLU*’s oncogenic impacts across many tumor types, which contributes to elucidating the probable mechanism of *CLU* in carcinogenesis and its clinical prognostic significance.

## Data Availability

The datasets used in this investigation are available in Internet repositories. The article/Supplementary Material contains the names of the repository/repositories and accession number(s).
