# Supplementary material for: A pan-cancer analysis reveals role of clusterin (CLU) in carcinogenesis and prognosis of human tumors
Source: Front Genet. 2023 Jan 4;13:1056184. doi: 10.3389/fgene.2022.1056184 (PMC9846084; doi:10.3389/fgene.2022.1056184)
Supplement: Supplementary file 1 [file Table1.DOCX]

Supplementary Material

# Supplementary Table

**Table S1.** gene expression data for 60 OSCC and 112 normal tissues from GEO database

| GSE | Total differentially  expressed genes | Up-regulated | Down-regulated | Technology/Platform | Sample size for each group |
| --- | --- | --- | --- | --- | --- |
| GSE138206 | 1756 | 972 | 784 | [HG-U133_Plus_2]  Affymetrix  Human Genome U133  Plus 2.0 Array | 6 cancer tissues 6 contralateral normal tissues 6 tissues adjacant to cancer |
| GSE13601 | 4230 | 1437 | 2793 | [HG_U95Av2] Affymetrix  Human Genome  U95 Version 2 Array | 31 cancer tissues 27 normal tissues |
| GSE31056 | 2459 | 1180 | 1279 | [HG-U133_Plus_2] Affymetrix  GeneChip Human Genome  HG-U133 Plus 2 Array  [Brainarray Version 12] | 23 cancer tissues 73 margin normal tissues |
| Intersection | 416 | 238 | 178 |  |  |

**Table S2.** Expression results of the final screened five differential genes in different data sets

| Gene |  | Log2FC |  |  | adj.P.Val |  |
| --- | --- | --- | --- | --- | --- | --- |
|  | GSE138206 | GSE13601 | GSE78060 | GSE138206 | GSE13601 | GSE78060 |
| CFD | -2.788600111 | -2.055809071 | -2.201918982 | 0.000187085 | 2.14E-09 | 0.009358046 |
| CLU | -2.260752461 | -1.727585949 | -3.768587542 | 0.000275858 | 0.000407028 | 0.011503766 |
| ABCA8 | -2.516874171 | -2.710794019 | -2.552601713 | 0.000571622 | 2.41E-07 | 0.04431405 |
| ADH1B | -2.844443838 | -3.16653475 | -3.702646599 | 0.002304717 | 3.82E-08 | 0.000672318 |
| CRISP3 | -4.87188203 | -2.625730345 | -5.767466592 | 0.006802578 | 9.80E-06 | 0.030553507 |

# Supplementary Figures


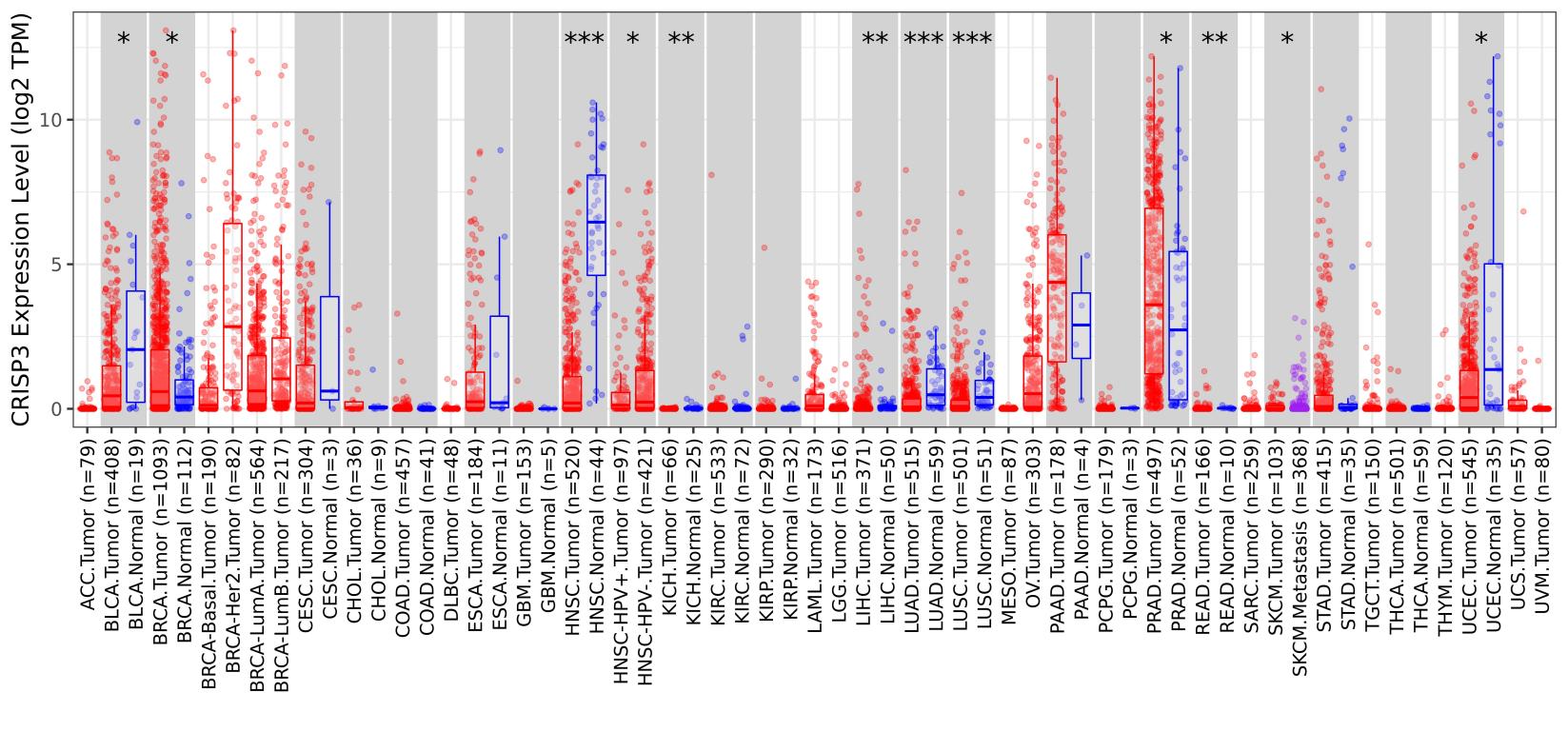


**Fig. S1. *CRISP3* expression levels in various tumor tissues and stages.** TIMER2 was utilized to examine *CRISP3* gene expression differences in different malignancies or tumor subtype tissues and nearby non-tumor tissues in the TCGA experiment. (**P <* 0.05; ***P <* 0.01; ****P <* 0.001)

**
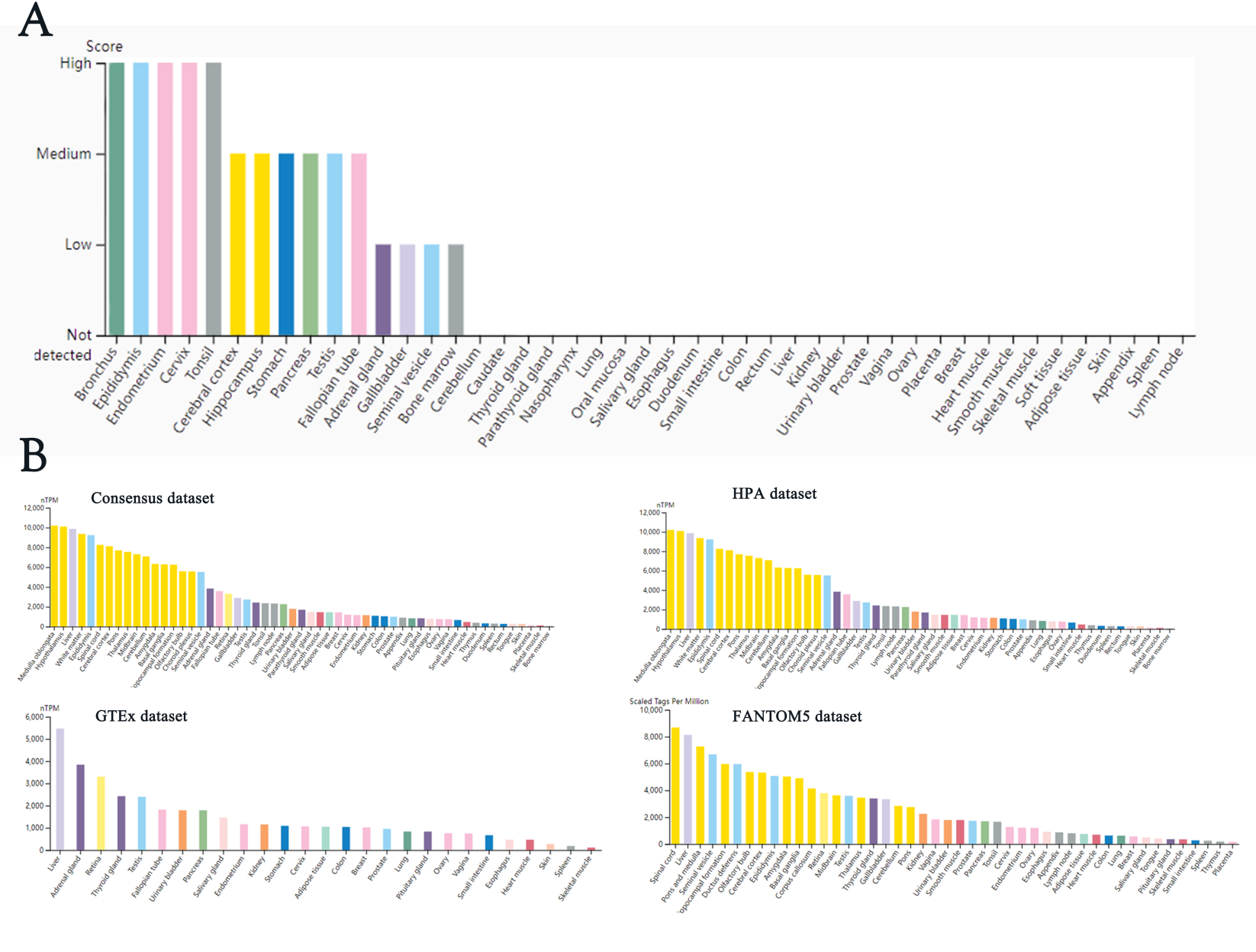
**

**Fig. S2. *CLU* expression levels in human tissues were obtained by analysis of HUMAN PROTEIN ATLAS tool** (A) *CLU* protein expression in various human tissues; (B) RNA expression of *CLU* in numerous human tissues.


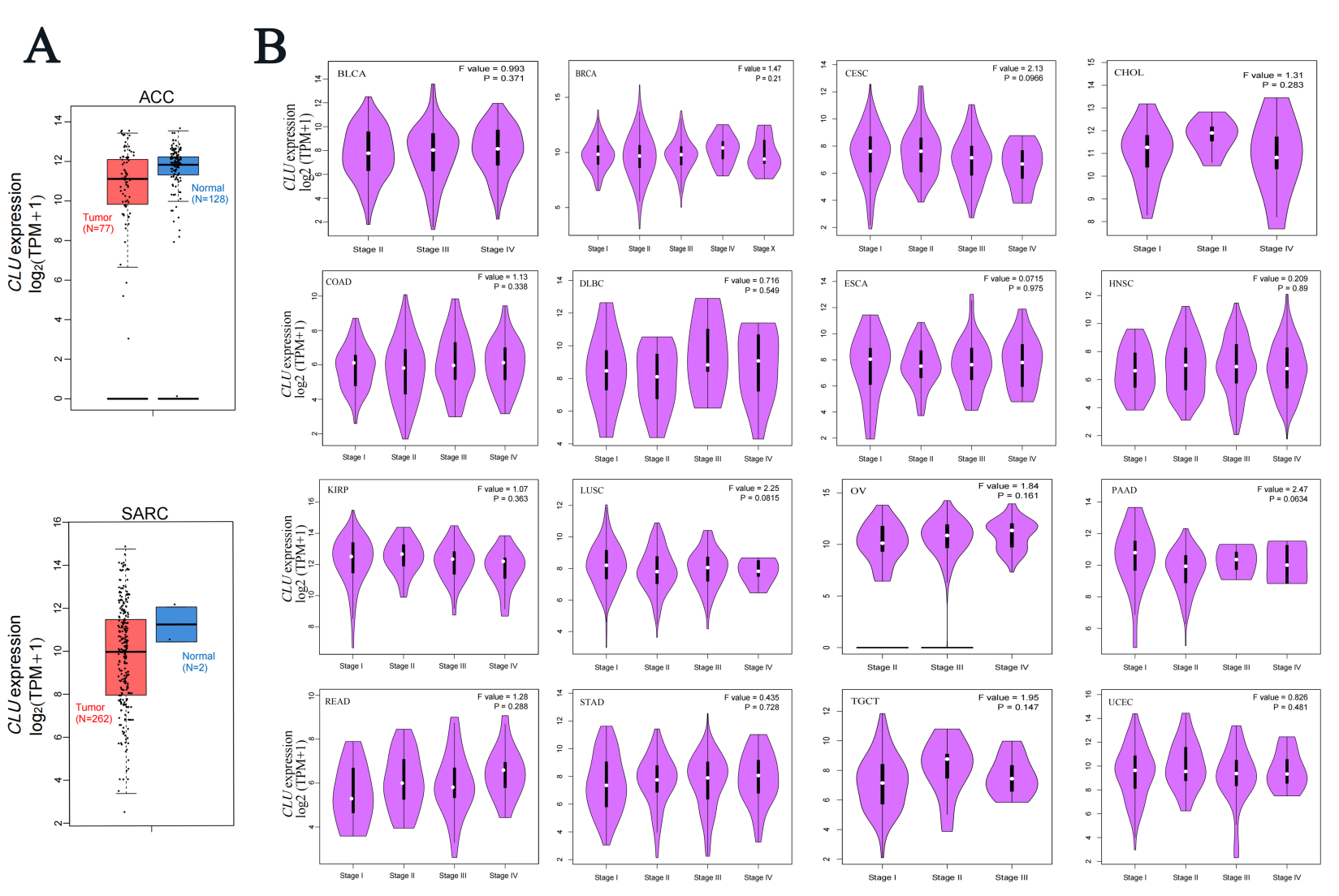


**Fig. S3. *CLU* gene expression levels in several types of cancers and pathological stages** (A) GTEx database's equivalent normal tissues were employed as controls, and GEPIA2 was utilized to examine the *CLU* gene expression status in ACC and SARC; (B) *CLU* expression levels by numerous pathological staging of BLCA, BRCA, CESC, CHOL, COAD, DLBC, ESCA, HNSC, KIRP, LUSC, OV, PAAD, READ, STAD, TGCT, and UCEC


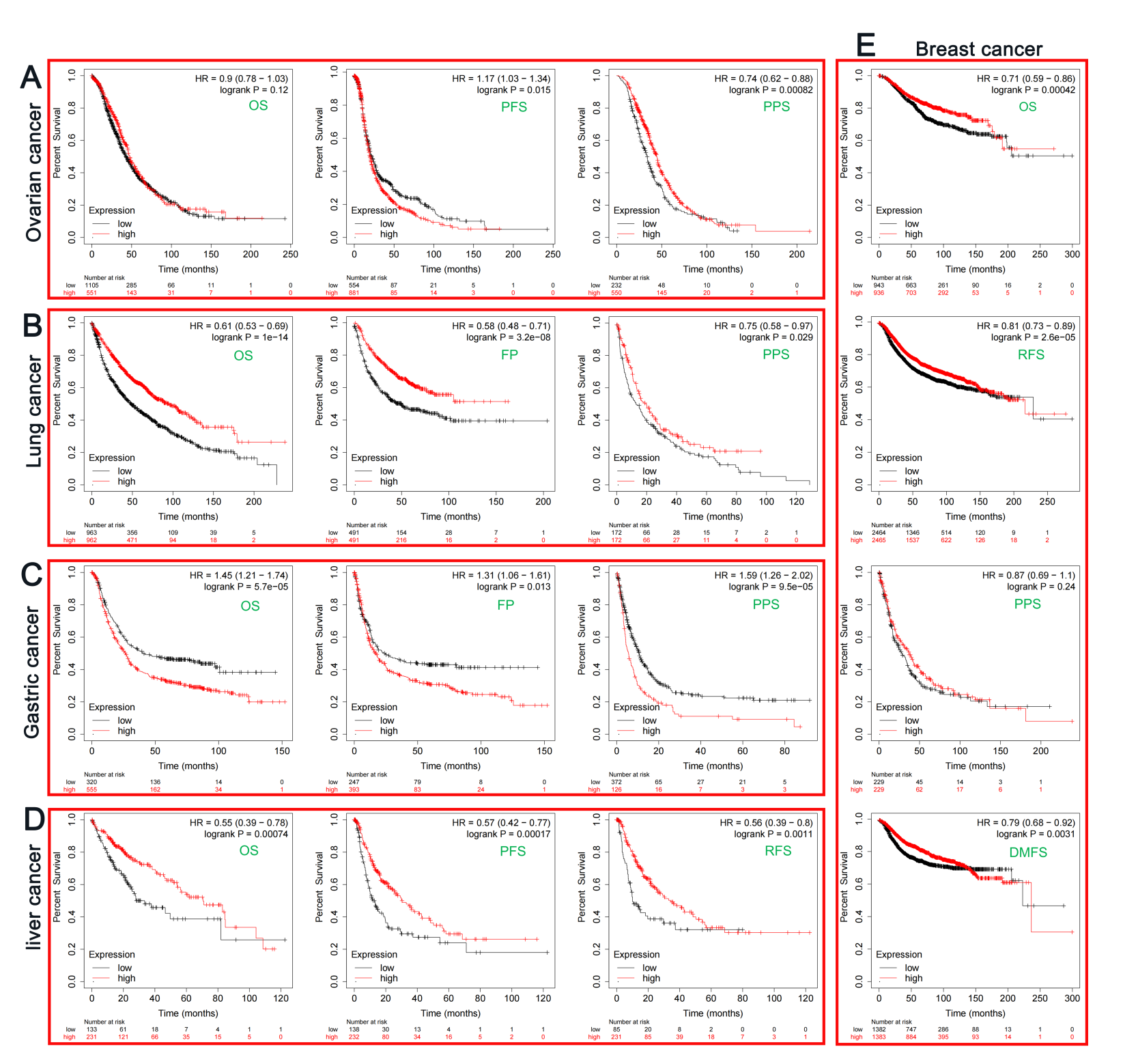


**Fig. S4. The link between *CLU* gene expression and cancer prognosis was investigated utilizing the Kaplan-Meier plotter.** We carried out a series of survival analyses utilizing Kaplan-Meier plotter depending on *CLU* expression level in ovarian cancer (A) lung cancer; (B) gastric cancer; (C) liver cancer (D); and BC; (E) cases, including OS, DMFS (Distant metastasis-free survival), RFS, PFS, PPS, and FP.


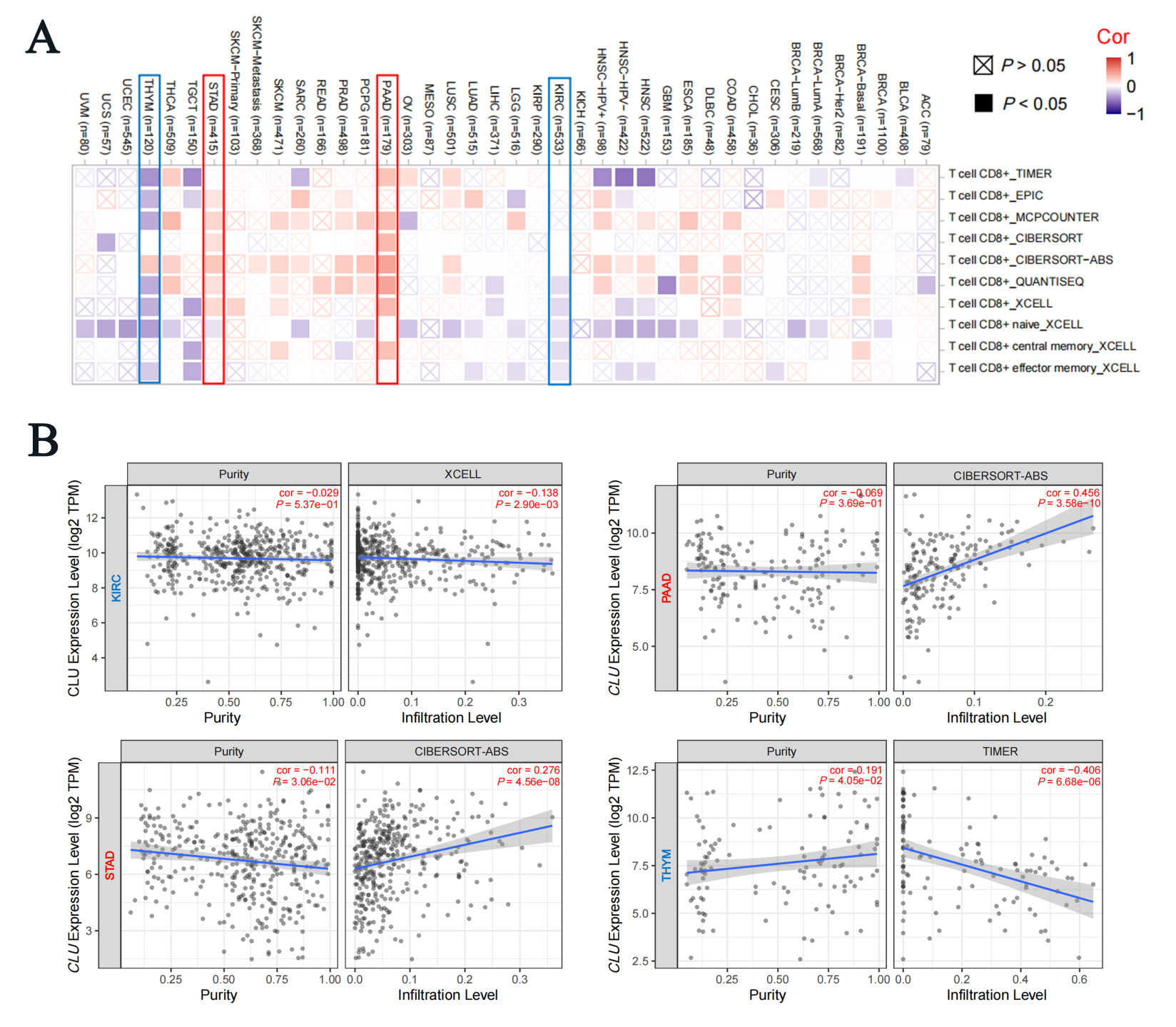


**Fig. S5. Correlation investigation of *CLU* gene expression with CD8+ T-cell immunological infiltration** (A) For all TCGA tumors, several algorithms (TIMER, EPIC, MCPCOUNTER, CIBERSORT, CIBERSORT-ABS, QUANTISEQ, and XCELL) were used to assess the connection between *CLU* expression and the amount of immunological infiltration of CD8+ T-cells; (B) scatterplot data of the selected tumor was provided, which was created using one of the methods.


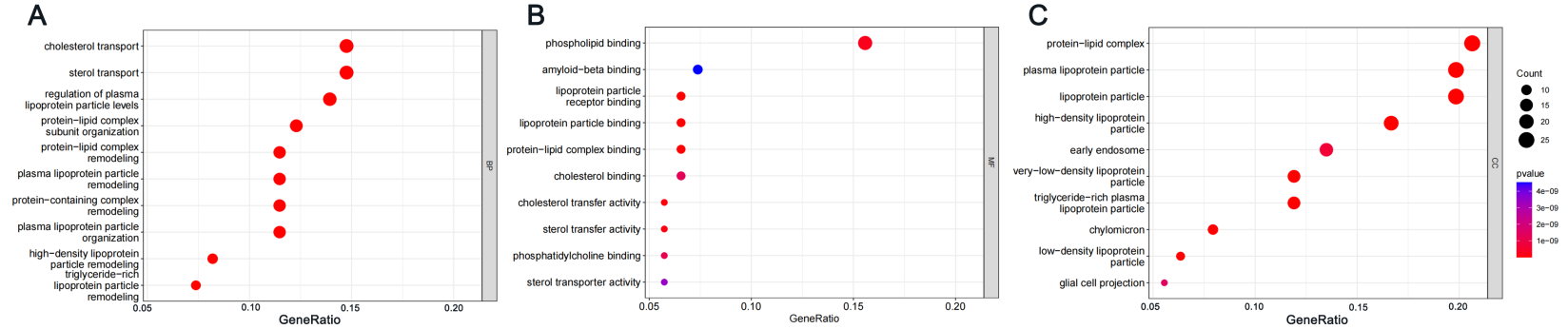


**Fig. S6. GO-biological process/molecular function/cellular component analysis of *CLU*-related genes.** (A) Dot plot for the biological process data in GO enrichment analysis is based on *CLU*-correlated genes and *CLU*-bound proteins; (B) dot plot for the molecular function data in GO enrichment analysis depends on *CLU*-correlated genes and *CLU*-bound protein; (C) dot plot for the cellular component data in GO analysis depending on *CLU*-correlated genes and *CLU*-bound protein.
